# The Effectiveness of Oral Health Education Among Non-dental Healthcare Professionals in Jeddah, Saudi Arabia: A Quasi-experimental Study

**DOI:** 10.7759/cureus.49187

**Published:** 2023-11-21

**Authors:** Dania Sabbahi

**Affiliations:** 1 Dental Public Health, King Abdulaziz University, Jeddah, SAU

**Keywords:** saudi arabia, applied medical sciences, nurses, physicians, oral health knowledge, oral health education

## Abstract

Objectives: The purpose of the study was to evaluate the level of oral health knowledge and practices among non-dental health professionals and to evaluate the effectiveness of oral health education provided to them.

Method: A convenience sample was recruited from non-dental healthcare professionals in Jeddah, Saudi Arabia. A self-administered questionnaire was distributed to participants to evaluate their oral health knowledge and behavior. Then, oral health educational material (a video) that was specifically developed for the study was sent to all participants to educate them about some oral health information related to adult oral health, oral signs of some systemic diseases, and the oral side effects of some medications. Two weeks later, the same questionnaire was sent to these participants to assess the effectiveness of the oral health education provided to them.

Result: The pre-intervention questionnaire results revealed a lack of oral health knowledge and inadequate oral health practices among the participants. After the intervention, the level of knowledge improved significantly from 6.4±2.2 to 10.4±3.8 out of 16 (p <0.001).

Conclusion: The oral health education intervention used in the current study was effective in improving oral health knowledge among non-dental healthcare professionals.

## Introduction

Oral health, which is an integral aspect of overall well-being, is connected to systemic health and quality of life [1]. Health cannot be optimal in the absence of oral health, as many medical conditions affect oral health and vice versa. One example is the impact of diabetes mellitus on the periodontium, which results in increased destruction of periodontal tissue in periodontitis patients. Moreover, periodontal disease undermines glycemic control in diabetic patients. This shows the bidirectional relationship between general and oral health [2].

Non-dental healthcare professionals, including physicians, nurses, and various allied healthcare professionals, are critical components of the healthcare system. Their interactions with patients occur in the context of a wide array of medical concerns, making them well-positioned to educate and advocate for oral health as an important component of holistic patient care [3]. Consequently, understanding the baseline knowledge and attitudes of non-dental healthcare professionals toward oral health is vital for a more comprehensive interdisciplinary approach to healthcare delivery.

Several studies have highlighted gaps in the oral health knowledge of non-dental healthcare professionals in Saudi Arabia [4-8]. This knowledge gap was attributed to the limited incorporation of oral health-related topics in medical, nursing, and allied healthcare undergraduate curricula and the limited dental continuous education available for non-dental healthcare professionals [9,10].

Several promising initiatives have attempted to bridge the knowledge gap among non-dental healthcare professionals and improve their oral health competence. These initiatives range from incorporating oral health topics into undergraduate curricula to implementing continuous education and interprofessional training programs [9,11-15].

A literature review found no report that evaluated such initiatives in Saudi Arabia. This study evaluated the oral health knowledge level and oral health practices of non-dental healthcare professionals and the effectiveness of the oral health education provided to them. The following null hypothesis was tested: there is no significant difference in oral health knowledge level before and after oral health education among non-dental healthcare professionals.

## Materials and methods

This study was approved by the research ethics committee of King Abdulaziz University, Faculty of Dentistry in Jeddah, Saudi Arabia (approval number #356-12-21).

A self-administered questionnaire was developed in English, consisting of 29 questions divided into three parts. The first part collected information about the participant’s characteristics, such as their age, gender, rank, field of specialty, years of experience, and practice location. The second part focused on the participant’s oral health practices, including brushing frequency and duration and the use of interdental cleaning aids and fluoridated products. The last part comprised 16 questions to assess oral health knowledge. The first five questions focused on the appearance of healthy and diseased periodontium and signs of gingivitis and periodontitis. The remaining questions tested the participants’ knowledge of the oral signs of different systemic diseases, the oral side effects of some medications, and the safest trimester to undergo elective dental treatment during pregnancy. This section is shown in Appendix A.

An oral health education video was developed specifically for this study. The video explained the relationship between systemic conditions and oral health, focusing on the relationship between diabetes and periodontal disease, oral signs of Addison’s disease, HIV, inflammatory bowel disease, anemia, the oral side effects of some medications, and pregnancy and dental care. This educational video is available upon request from the author.

The study recruited a convenience sample of non-dental healthcare professionals from different hospitals in Jeddah, Saudi Arabia. Two dental interns visited different government and private hospitals, as well as primary healthcare centers and polyclinics, between January and April 2022 to recruit non-dental healthcare professionals, including students, interns, postgraduates, specialists, and consultants. Attention was directed to selecting practitioners with different specialties, including medicine, nursing, and the allied medical sciences.

The questionnaire was administered online using Google Forms (Google Inc., Mountain View, CA). All participants consented electronically before completing the online questionnaire. Then, they were sent the oral health education video. Two weeks later, the participants’ oral health knowledge was reassessed with the third section of the questionnaire.

The sample size was calculated using an online calculator, which indicated that 128 participants were needed to achieve a power of 80% and to detect an effect size of 0.25 at the 5% significance level (two-sided) [16].

The data were collected and tabulated, and descriptive statistics (frequencies and percentages for categorical variables and mean, median, and standard deviation for quantitative variables) were calculated. The total pre-and post-test scores were computed by assigning one point for each correct answer. The normality of both the pre-and post-test scores was assessed. Non-parametric tests were selected because neither score was normally distributed. McNemar’s test was used to compare the percentages of correct answers between the pre-and post-tests. The Wilcoxon signed-rank test was used to compare the pre-and post-test scores. The Kruskal-Wallis test was used to compare the change between the post- and pre-test scores among specialties. All statistical analyses were two-tailed and conducted using IBM SPSS software for Windows (Ver. 23.0; IBM Corporation, Armonk, NY) at a significance level of 0.05.

## Results

Sample characteristics

The study recruited 165 participants, of whom 127 completed both the pre- and post-questionnaires. Among the participants, 53% (N=89) were female, and 56% (N=93) of them were interns or students. Most of the participants were in the medical field (49.1%, N=81), followed by nursing (36.4%, N=60). The majority of the participants (72.1%, N=119) had <5 years of experience; 74.5% (N=123) worked in the government sector, 12.1% (N=20) in primary healthcare centers, and the rest in private hospitals/clinics (Table 1).

**Table 1:** Characteristics of the sample (N=165) The data have been represented as N and %

Variable			Count (N)	%
Gender	Female		89	53.9%
	Male		76	46.1%
Rank	Student	Medicine	21	20.0%
		Nursing	8	
		Allied medical sciences	4	
	Intern	Medicine	26	36.4%
		Nursing	27	
		Allied medical sciences	7	
	Postgraduate student	Medicine	20	16.4%
		Nursing	2	
		Allied medical sciences	5	
	General practitioner		2	1.2%
	Nurse		19	11.5%
	Specialist		20	12.1%
	Consultant		4	2.4%
The field of specialty	Medicine		81	49.1%
	Nursing		60	36.4%
	Allied medical sciences		24	14.5%
Professional experience	Less than 5 years		119	72.1%
	5-10 years		32	19.4%
	11-20 years		12	7.3%
	More than 20 years		2	1.2%
Main location of practice	Governmental hospitals		123	74.5%
	Private sector		19	11.5%
	Primary health care center		20	12.1%

Oral health practices

**Figure 1:**
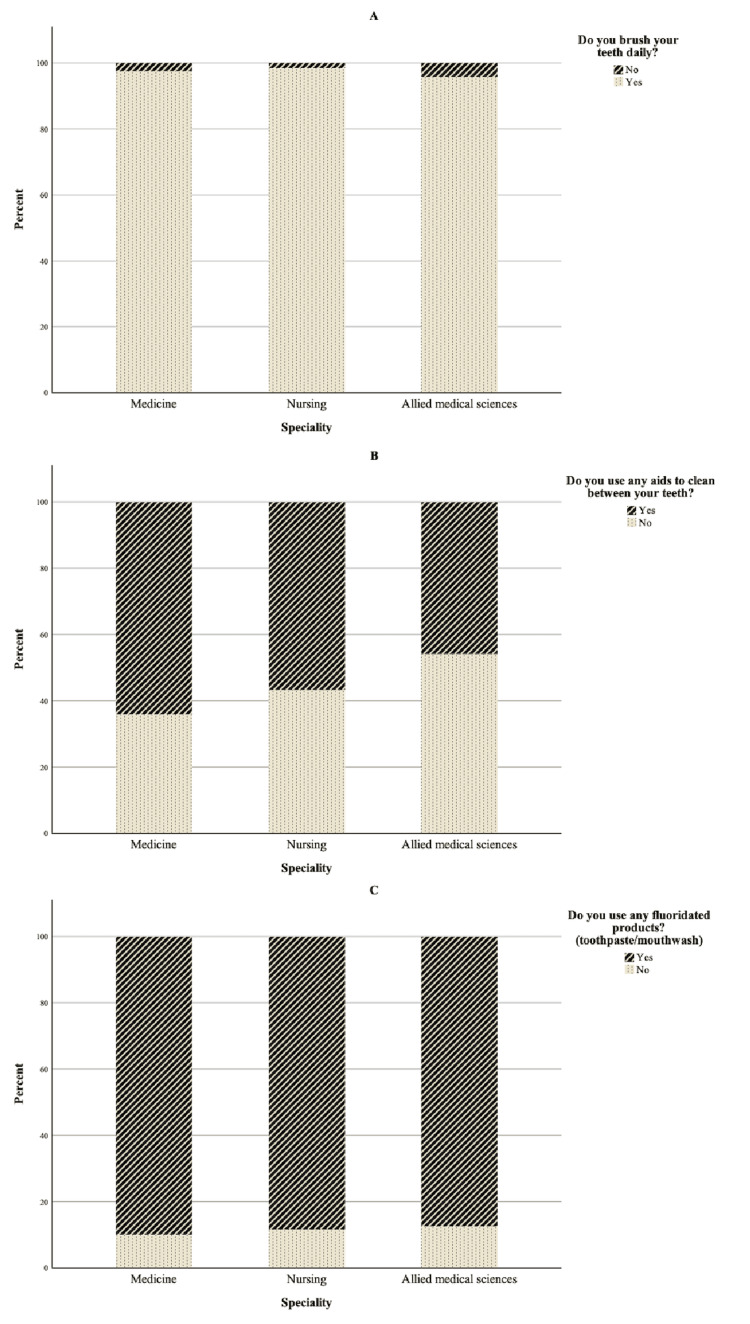
(A) Bar chart for brushing; (B) usage of interdental cleaning aids; (C) usage of fluoridated products among different specialties The data have been represented as %

When asked about their oral health practices, 97.6% (N=161) of the sample stated that they brushed their teeth daily, with 69.1% (N=114) brushing twice a day. The average duration of brushing among the participants was >2 minutes, 1-2 minutes, and <1 minute for 10.3% (N=17), 47.3% (N=78), and 42.4% (N=70) of the participants, respectively. The majority of the sample (89.1%, N=147) used a fluoridated product (toothpaste/mouthwash). More than half (58.8%, N=97) used interdental cleaning aids, such as dental floss (40.2%, N=39), a water flosser (35.1%, N=35), and toothpicks (23.7%, N=23). Figure 1 plots the oral health behavior data of the sample.

Pre-intervention knowledge

In the pre-intervention questionnaire, the majority of the participants recognized the pictures of healthy gingiva, gingivitis, and periodontitis (97.6% (N=161), 87.3% (N=144), and 49.1% (N=81), respectively). Only 27.3% (N=45) recognized bleeding as the main sign of gingivitis, and 30.3% (N=50) recognized bone loss as the main sign of periodontitis. About one-third (35.8%, N=59) were able to identify the relationship between diabetes and gingival health, and about 45% (N=75) of them recognized hairy leukoplakia at the lateral border of the tongue as a common mucosal lesion associated with HIV. Only a minority recognized pyostomatitis vegetans as a sign of inflammatory bowel disease (17%, N=28) and glossitis as an oral sign of anemia (12%, N=20). When asked about the oral lesions associated with some medications, 61.2% (N=101) and 50.9% (N=84) of the participants recognized the medications associated with gingival hyperplasia and angioedema, respectively. When asked about the management of medication-induced xerostomia, only 16.4% (N=27) answered correctly. Lastly, about one-quarter of the sample (29.1%, N=48) were aware of the safest trimester for elective dental treatment during pregnancy.

Post-intervention knowledge

The percentage of correct answers improved significantly for 11 of the 16 questions in the post-intervention knowledge questionnaire (Table 2).

**Table 2:** Percentages of the correct answers for the questions in the pre- and post-test scores (n=127) The data have been represented as % *Using McNemar's test, the value at which the p-value is considered significant is (p<0.05).

Question	Percentage of correct answers		p-value*
	Pre-test	Post-test	
Recognizing a picture of healthy periodontium	97.6%	99.2%	1.00
Recognizing a picture of gingivitis	87.3%	98.4%	0.006
Recognizing a picture of periodontitis	49.1%	66.1%	0.015
Recognizing the diagnostic signs of gingivitis	27.3%	29.1%	0.850
Recognizing the diagnostic signs of periodontitis	30.3%	37.0%	0.296
Recognizing the relationship between diabetes and periodontal disease	35.8%	76.4%	<0.001
Recognizing a picture of the oral hyperpigmentation as a sign of Addison’s disease	28.5%	33.1%	0.180
Recognizing a picture of a hairy leukoplakia at the lateral border of the tongue as a common oral lesion associated with HIV	45.5%	80.3%	<0.001
Recognizing pyostomatitis vegetans as an oral sign of inflammatory bowel disease	17.0%	70.1%	<0.001
Recognizing glossitis as a sign of anemia	12.1%	82.7%	<0.001
Recognizing the oral signs that might be associated with systemic sclerosis (scleroderma)	25.5%	37.8%	<0.001
Recognizing the medication that might be associated with gingival hyperplasia	61.2%	74.8%	<0.001
Recognizing angioedema	9.7%	72.4%	<0.001
Recognizing the medication associated with angioedema	50.9%	79.5%	<0.001
Management of medication-induced xerostomia	16.4%	19.7%	0.125
Recognizing the safest trimester for elective dental treatment during pregnancy	29.1%	80.3%	<0.001

Overall, the mean knowledge score improved from 6.4 (SD = 2.2) to 10.4 (SD = 3.8). The Wilcoxon signed-rank test revealed a significant improvement in oral health knowledge (p<0.001; Table 3).

**Table 3:** Comparison between the pre- and post-intervention knowledge test scores (n=127) The data have been represented as mean, median, and standard deviation *The value at which the p-value is considered significant (p<0.05).

	Pre-test total score	Post-test total score
Mean	6.4	10.4
Median	7	11
Standard deviation	2.2	2.8
P-value for Wilcoxon signed-rank test	<0.001*	

**Figure 2:**
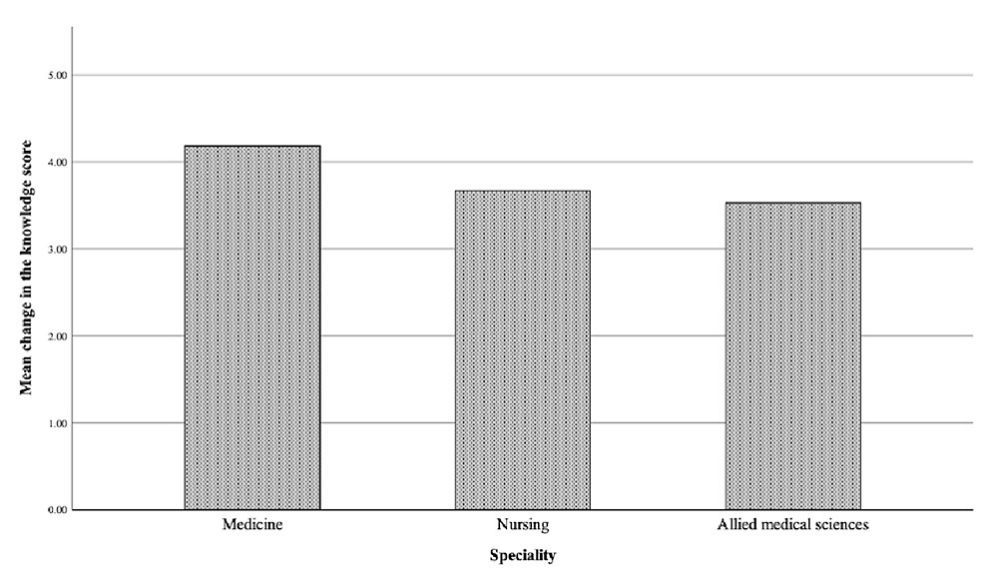
Bar chart for the changes in the knowledge scores among different specialties The data have been represented as mean

The Kruskal-Wallis test did not show any significant difference in the pre-or post-test results among the different specialties (p=0.408; Figure 2).

## Discussion

This is one of the first studies to assess the effectiveness of oral health education for non-dental healthcare professionals in Saudi Arabia. The study revealed that the participants had limited baseline oral health knowledge. This is in agreement with published studies conducted in Saudi Arabia [4-8] and internationally [17-23]. Unlike most of these previous studies, which targeted pediatricians and focused on assessing knowledge related to child oral health, the current study focused on assessing knowledge related to the oral health of adults, oral signs of systemic diseases, and oral side effects of some medications. The questionnaire focused on systemic diseases and medications that are commonly encountered by general practitioners and consultants within specialties related to family and internal medicine.

The educational intervention had a positive impact, with a significant improvement seen in the post-intervention knowledge score. Numerous studies have demonstrated similar positive impacts of educational interventions on the oral health knowledge of non-dental healthcare professionals [9,11-15]. These efforts to bridge the knowledge gap among non-dental healthcare professionals have included continuing education programs, interprofessional training, and the integration of oral health components into the undergraduate and postgraduate curricula.

In the present study, an educational video was used to deliver oral health information to the participants. This is a convenient, accessible, and cost-effective way to promote positive change and improvement in patient behavior in health promotion research [24-26]. This strategy also accords with the preference of general practitioners for short online on-demand options, including videos, podcasts, and written material [27,28].

While educational interventions can enhance oral health knowledge, ensuring the sustainability of the knowledge remains a challenge [13]. Several key areas should be tackled to ensure sustainability and implementation in practice, including ongoing continuous education and training programs to refresh oral health knowledge among non-dental healthcare professionals and encourage interdisciplinary collaboration for a holistic approach to patient care. Future long-term studies are required to assess the sustainability of the provided knowledge and to examine various strategies that can facilitate its attainment.

Non-dental healthcare professionals who receive structured oral health education are more likely to identify oral health issues early, refer patients to dental professionals when necessary, and provide appropriate preventive advice [29]. Non-dental healthcare providers may face barriers such as time constraints, competing healthcare priorities, and a lack of readily available dental resources for referrals [30]. The current study did not assess the impact of the acquired knowledge on the participants’ practices. This can be the focus of future studies to evaluate the impact of oral health education on the daily practice of non-dental healthcare professionals.

The present study has some limitations. The first arises from convenience sampling which has the potential to introduce selection bias and could impact the generalizability of the findings. Additionally, the study results are based on self-reports and may be susceptible to self-report bias. Another limitation relates to the nature of the questions asked. About 23% (N=38) of the participants in some medical specialties and allied medical sciences did not finish the post-intervention questionnaire because they felt that the educational content provided was irrelevant to their practice.

The knowledge gap observed in this study deserves attention from the healthcare community. There is a need to develop tailored continuing education materials to provide relevant educational content to different non-dental healthcare professionals. These educational efforts should be designed to reach healthcare professionals in their workplaces and fit within their busy schedules. In addition, a variety of educational strategies and materials should be used that consider the preferences of healthcare professionals. Initiatives to develop educational resources and make them available to different healthcare professionals are strongly recommended. These resources will be of great help to all non-dental healthcare professionals who want to educate themselves and can be used by dental healthcare professionals to educate their non-dental colleagues in their workplaces. Additionally, policymakers and professionals across various healthcare disciplines should collaborate in order to integrate oral health components into undergraduate curricula for non-dental healthcare programs.

## Conclusions

This study showed inadequate oral health behaviors among some of the participants. Specifically, 30.1% of the sample reported insufficient brushing frequency (less than twice a day), 42.2% reported an average brushing duration of less than two minutes, and 41.2% reported a lack of utilization of interdental cleaning aids. In addition, a low level of oral health knowledge was noticed among the participants, with a mean pre-intervention knowledge score of 6.4 out of 16. The educational intervention used in the current study resulted in a significant improvement in oral health knowledge among the participants. This study can be the nidus for tailored educational interventions to improve the knowledge level among non-dental healthcare workers in the Kingdom of Saudi Arabia.

## Appendices

Appendix A 

**Table 4:** Oral health knowledge questionnaire

No.	Question	Notes
Q1	Which one of the following pictures represents a healthy gum?	3 pictures were presented to choose from them
Q2	Which one of the following pictures represents an inflamed gum?	3 pictures were presented to choose from them
Q3	Which one of the following pictures represents periodontitis?	3 pictures were presented to choose from them
Q4	Which of the following signs represents a diagnostic sign of gingivitis (inflammation in the gum)? Erythema, Bleeding, Halitosis, Gum pain	
Q5	Which of the following signs represents a diagnostic sign of periodontitis? Swelling, Bone loss, Erythema, Toothache	
Q6	A 44-year-old diabetic male patient came to your clinic complaining of teeth sensitivity, bad odor, and teeth mobility. After clinical and radiographic diagnosis this patient was diagnosed with periodontitis stage III grade C. Regarding the mentioned scenario which of the following is/are the right answers? There is no relation between high levels of HbA1c and periodontitis. A high level of HbA1c will worsen the periodontitis. Periodontitis will inhibit HbA1c control B and C	
Q7	Which of the following pictures indicates the oral sign of Addison's disease?	2 pictures were presented: a picture of a hyperpigmented lower lip and a picture of a swollen depapillated tongue (glossitis)
Q8	This oral mucosal lesion is commonly associated with? HPV, HIV, HSV, Coxsackie virus	A picture of oral hairy leukoplakia on the lateral border of the tongue was presented.
Q9	The following picture is of a patient with an oral lesion called pyostomatitis vegetans. Which disease does this oral lesion is associated with? Cushing syndrome, Inflammatory bowel disease, Sjögren's Syndrome, Hashimoto’s thyroiditis	
Q10	A 25-year-old female patient presented to your clinic with tongue tongue-burning sensation. She is healthy but on a diet to lose weight. What will you suspect? Anemia, Vitamin B12 deficiency, Dehydration, Vitamin C deficiency	A picture of swollen depapillated tongue (glossitis) was presented.
Q11	Which of the following is an oral sign that might be associated with systemic sclerosis (scleroderma)? Limited mouth opening and xerostomia, Limited mouth opening and hypersalivation, Limited mouth opening only.	
Q12	What is the medication that might cause this lesion? Metformin, Phenytoin, Tetracycline, Ibuprofen	A picture of severe gingival hyperplasia was presented
Q13	What is this phenomenon/lesion called? Lip edema, Angioedema, Reaction to lip filler, Granulosis	
Q14	This phenomenon in the previous question is one of the complications of one of the blood pressure drug classes, which one is it? Diuretics, ACE inhibitors, Beta blockers, Calcium channel blocker	
Q15	A 24 male patient came to your clinic complaining of severe dryness of the mouth and difficulty in swallowing, he mentioned that he has been on antidepressants for 6 months. What will be your management? Stop the medication, Change the type of medication, Mouthwash prescription, Advise him to a dental check-up, Assure the patient, and follow up every 3 months.	
Q16	In which trimester should you refer a pregnant woman safely for an elective dental treatment? 1^st^ trimester, 2^nd^ trimester, 3^rd^ trimester	
